# Environmental Filtering Drives the Assembly of Habitat Generalists and Specialists in the Coastal Sand Microbial Communities of Southern China

**DOI:** 10.3390/microorganisms7120598

**Published:** 2019-11-21

**Authors:** Anyi Hu, Hongjie Wang, Meixian Cao, Azhar Rashid, Mingfeng Li, Chang-Ping Yu

**Affiliations:** 1 CAS Key Laboratory of Urban pollutant Conversion, Institute of Urban Environment, Chinese Academy of Sciences, Xiamen 361021, China; hjwang@iue.ac.cn (H.W.); mxcao@iue.ac.cn (M.C.); azoo74@yahoo.com (A.R.); cpyu@iue.ac.cn (C.-P.Y.); 2 University of Chinese Academy of Sciences, Beijing 100049, China; 3 Nuclear Institute for Food and Agriculture, Tarnab, Peshawar 446, Pakistan; 4 School of Biological Science and Biotechnology, Minnan Normal University, Zhangzhou 363000, China; 5 Graduate Institute of Environmental Engineering, National Taiwan University, Taipei 106, Taiwan

**Keywords:** microbial community, habitat generalists and specialists, environmental filtering, random dispersal, intertidal and supratidal sands

## Abstract

Coastal sands harbor diverse microbial assemblages that play a critical role in the biogeochemical cycling of beach ecosystems. However, little is known about the relative importance of the different ecological processes underlying the assembly of communities of sand microbiota. Here, we employed 16S rDNA amplicon sequencing to investigate the sand microbiota of two coastal beaches, in southern China. The results showed that sand microbial assemblages at intertidal and supratidal zones exhibited contrasting compositions that can be attributed to environmental filtering by electric conductivity. A consistent pattern of habitat generalists and specialists of sand microbiota was observed among different beach zones. Null and neutral model analyses indicated that the environmental filtering was mainly responsible for supratidal microbial communities, while the neutral processes could partially influence the assembly of intertidal communities. Moreover, environmental filtering was found to shape the habitat specialists, while random dispersal played a major role in shaping generalists. The neutral model analysis revealed that the habitat generalists exceeding the neutral prediction harbored a relatively higher proportion of microbial taxa than the specialist counterparts. An opposite pattern was observed for taxa falling below the neutral prediction. Collectively, these findings offer a novel insight into the assembly mechanisms of coastal sand microbiota.

## 1. Introduction

Sandy beaches are one of the most important components of the world’s unfrozen shoreline [1,2]. The biofilm microorganisms attached to the sand surfaces play an essential role in mediating the biogeochemical cycling of major elements (e.g., carbon, nitrogen and sulfur etc.), and the removal of pollutants in sand ecosystems [3,4]. Furthermore, the indigenous microbial sand communities are also one of the most critical factors for preventing the colonization of harmful exogenous microorganisms, such as fecal indicator bacteria and pathogens, on beach sands [5,6]. Thus, increasing attention is being paid to the biogeographic distribution pattern of sand microbial communities and its driving forces [2,4,7,8,9]. The importance of environmental filtering in shaping the assembly of sand microbial communities has highlighted, and a significant body of literature has demonstrated, that the distance from shoreline, sand grain size, moisture, temperature, nutrients (including organic and inorganic matters), and chemical contaminants are major environmental factors shaping sand microbial communities [2,7,8,10,11]. Therefore, sand microbial assemblages have been proposed as a sensitive bio-indicator for evaluating the environmental health of coastal habitats [10,12]. In this regard, geographic distance has also been found to play a certain role in controlling the spatial distribution of sand microbial assemblages, suggesting that dispersal limitations may affect sand microbial communities as well [8,13]. In addition, disturbances like wave- or tidal-action may lead to the random dispersal of microbes between seawater and intertidal sands, or across the unsaturated zone of beach aquifers [2,14,15]. However, little is known about the relative importance of different ecological processes (e.g., environmental filtering and dispersal-related mechanisms) in determining the assembly of sand microbial communities, and their key ecological groups (e.g., habitat generalists and specialists).

Mounting evidences suggest that microbial species can be partitioned into habitat generalists and specialists, on the basis of their distinct capacities to adapt to wide ranging environmental conditions [16,17]. The habitat generalists have broad environmental tolerances and can occur in many habitats, whereas the habitat specialists are more restricted to specific habitats, due to their narrow environmental tolerances [18,19]. Hence, the habitat specialists are more vulnerable and susceptible to extinction under changing environmental conditions than the habitat generalists [20]. As such, the assembly of habitat generalists and the specialists are controlled by different ecological processes [17,21,22]. For example, Pandit and colleagues found that dispersal-related mechanisms and environmental filtering were responsible for the assembly of habitat generalists and specialists of zooplankton communities in coastal rock pools, respectively [18]. A similar pattern was also observed for lake microbial communities, where the assembly of habitat generalists was, to a greater extent, shaped by neutral processes, as compared to habitat specialists [22]. However, the results of some recent studies indicate that the habitat generalists in the microbial communities may be more sensitive to changes in environmental conditions than habitat specialists [17,23]. In view of these controversial findings, it remains an open question as to how environmental filtering and dispersal-related ecological processes may affect the distribution of habitat generalists and specialists of the microbial communities in diverse environments. Furthermore, little is known about the assembly mechanisms of key ecological groups of sand microbial assemblages. Answering these questions will be key to better understand and predict the fate of sand microbiota, in the current context of global climate change [24]. 

In the present study, the biogeographic distribution of sand microbial communities was investigated and their key ecological groups (i.e., habitat generalists-specialists) were identified from the intertidal and supratidal zones of Dongshan Island, Fujian Province, China, by using 16S rDNA amplicon sequencing. The relative importance of different ecological processes in the microbial community assembly was investigated by employing a β-diversity null model test [25], neutral model test [26], and multivariate statistical analysis [27]. Given the different hydrological dynamics in the intertidal (frequent tidal inundation) and supratidal (no tidal inundation) zones [3], we hypothesized that: (i) there is a significant difference in the composition of microbial communities from the intertidal and supratidal zones, where environmental filtering is the main driving force causing such differences; (ii) random dispersal would play a more important role in determining the microbial assemblages in the intertidal zones than supratidal zones; and (iii) whether the second hypothesis is verified or not, habitat specialists are, to a greater extent, assembled by environmental filtering as compared to habitat generalists. Since habitat generalists may have a stronger capacity to tolerate a range of environmental conditions of the coastal sands, as compared to habitat specialists.

## 2. Materials and Methods

### 2.1. Sampling Area, Sampling Collection and Measurement of Environmental Parameters

**Figure 1:**
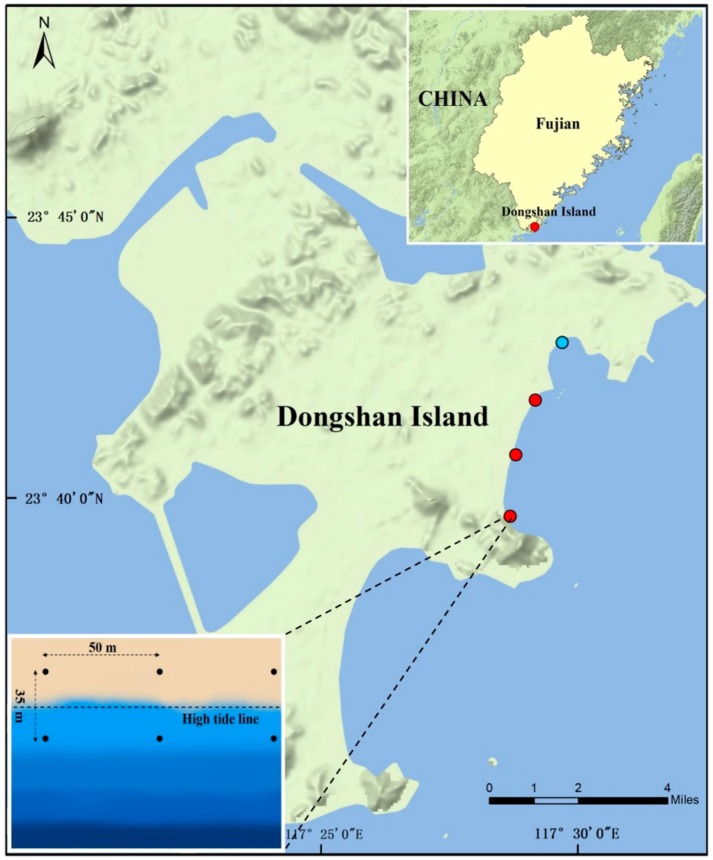
The location of sampling sites in Dongshan Island, Fujian Province, China. Three of four sites (red dots) located in Jinluan Bay, while another one (blue dot) located in Maluan Bay. Triplicate surface sediments (0–10cm) were collected from supratidal or intertidal zones of each site.

Dongshan Island is one of the most famous coastal resorts in Fujian Province, China (Figure 1). A total of 24 surface sandy sediment samples (0–10 cm) were collected in triplicate at supratidal and intertidal zones, from three sampling sites at Jinluan Bay, and one site at Maluan Bay of Dongshang Island, on 11 July 2017 (Figure 1). The sediment samples were shipped to the laboratory in iceboxes within 6 h of sampling, and stored at −80°C until further analysis.

The physico-chemical properties of the sediment samples were characterized as described in our previous work [28]. Briefly, the sediment grain size was measured using a laser scattering particle analyzer (MS2000, Malvern Instruments, Malvern, UK). Sediment pH was determined by UB-7 Ultra Basic pH-detection (Denver Instruments, Arvada, CO, USA), with a sediment to water ratio of 1 to 2.5 (*w*/*w*), and electric conductivity (EC) was measured by a WTW multi 340i conductivity meter (WTW, Weilheim, Germany), with a sediment to water ratio of 1 to 5 (*w*/*w*). Total organic carbon (TOC) and total nitrogen (TN) were determined by a TOC analyzer (Shimadu, Japan). The NH_4_-N, NO_2_-N and NO_3_-N were extracted from the sediment samples using 2 M KCl, and then determined by a Lachat QC8500 Flow Injection Autoanalyzer (Lachat Instruments, Loveland, Colorado, USA). The available phosphorus (AP) was measured by the sodium bicarbonate extraction method [29]. The concentrations of eight heavy metals including chromium (Cr), nickel (Ni), copper (Cu), zinc (Zn), cadmium (Cd), lead (Pb), mercury (Hg), and Arsenic (As) were determined by an Agilent 7500cx ICP-MS (Agilent Technologies, Santa Clara, CA, USA) [21].

### 2.2. DNA Extraction, PCR Amplification and 16S rDNA Amplicon Sequencing

DNA was extracted from ~0.3 g sandy sediments by using the FastDNA SPIN Kit for Soil (Qbiogene-MP Biomedicals, Irvine, CA, USA), according to the manufacturer’s instructions [27]. The universal primer pair 515F (5’-GTG YCA GCM GCC GCG GTA-3’) and 907R (5’-CCG YCA ATT YMT TTR AGT TT-3’) were used to amplify the V4–V5 region of the microbial 16S rRNA genes [30]. Each PCR reaction was performed in a 25 μL reaction volume, consisting of 12.5 μL of the AmpliTaq™ Gold PCR Master Mix (2×) (Applied Bio-systems, Foster, CA, USA), 0.4 μM of each primer, and ~20 ng DNA template. The PCR amplification was programed as follows: an initial denaturation at 95 °C for 5 min, followed by 25 cycles of 95 °C for 30 s, 55 °C for 30 s, and 72 °C for 90 s, and a final extension at 72 °C for 10 min. The PCR products were purified and quantified as described previously [27]. The purified PCR products were sequenced on the Illumina MiSeq platform (Illumina Inc., San Diego, CA, USA) with paired-ends (2 × 300 bp). The raw sequence data was deposited in the NCBI short reads archive database under BioProject number PRJNA578248.

### 2.3. Sequence Analysis

The raw paired-end reads were denoised and assembled using DADA2 v1.1.3 [31] by following the pipeline described in https://benjjneb.github.io/dada2/tutorial.html. The high-quality reads were then clustered into amplicon sequence variants (ASVs) based on 100% identity. The taxonomic assignment was performed by using the RDP classifier and the SILVA database (v132) (https://www.arb-silva.de/) [32], with a confidence threshold of 80%. All samples were randomly sub-sampled to the smallest library size (19,000 reads) in order to standardize the uneven sequencing effort. 

### 2.4. Statistical Analysis

Principal component analysis (PCA) was used to identify the variation of environmental variables of the coastal sandy sediments. Non-metric multidimensional scaling (NMDS) was used to summarize patterns of microbial community structure based on Bray-Curtis distance matrices. Permutational multivariate analysis of variance (Adonis) and analysis of similarity (ANOSIM) were used to test the significance of the differences between supratidal and intertidal sand microbial communities [27]. 

The relationship between the microbial community composition and environmental factors was explored by using a constrained analysis of principal coordinates (CAP) [27]. Prior to CAP analysis, multicollinearity between environmental variables was assessed by using Variation Inflation Factor (VIF), using the ‘vif’ function in the R package usdm 1.1-18 (https://cran.r-project.org/web/packages/usdm/index.html). Any variables with high multicollinearity (i.e., variables with VIF values >5) were excluded from the further analysis [33]. Since, spatial variables can be used as proxies for the unmeasured environmental variables or dispersal limitation [34], the partial distance-based redundancy analysis (partial db-RDA) was used to further distinguish the relative contribution of environmental and spatial factors on the variation of microbial community compositions. Principal Coordinates of Neighborhood Matrices (PCNM) were used as spatial variables for the partial db-RDA analysis [35]. The geographic coordinates were transformed into a matrix of spatial vectors using the ‘PCNM’ function in vegan v2.5-3 [36]. A forward selection method was used to identify the best environmental and spatial variables explaining community variation by using the ‘ordiR2step’ function in the vegan (v2.5-3) R package [36].

The habitat generalists and specialists of the microbial communities from coastal sandy sediments were identified based on the Levins’ niche breadth (B) index with permutation algorithms (1000 permutations) by using EcolUtils v0.1 (https://github.com/GuillemSalazar/EcolUtils). In addition, the indicator genera for supratidal and intertidal communities were classified by using the ‘IndVal’ function in the labdsv (v1.8-0) R package [37]. Only, the genera with highly significant indicator values (IndVal value > 0.75, *p* < 0.01) were considered as good indicators for either environments (i.e., supratidal or intertidal). All statistical analysis and visualization were performed using R (v3.6.0) with the packages phyloseq (v1.28.0) [38], ggplot2 (v3.2.0) [39], and ComplexHeatmap (v1.20.0) [40]. All R codes used in this study are available online (https://github.com/AnyiHu/Coastal_sand_microbiota).

### 2.5. Null Model and Neutral Model Analysis

The abundance-based diversity null model was used to evaluate the relative importance of niche and neutral processes on the assembly of the sand microbial communities, as described previously [25]. A null distribution of the expected β-diversity was generated on the basis of 1000 random shuffles of the original community data. Then, a β-null deviation was calculated as difference between the observed mean β-diversity and the expected mean β-diversity [25]. Furthermore, the Sloan neutral model was applied to evaluate the effects of random dispersal and ecological drift on the assembly of sand microbial communities [26,41]. This model was fit to the occurrence frequency of ASVs and their mean relative abundance in the metacommunity by a single free parameter describing the migration rate (m) [26]. The estimated m represents the probability of random loss of an individual in a local community replaced by dispersal from the metacommunity, and can thus be interpreted as a measure of dispersal limitation. A high m value indicates lower dispersal limited microbial communities. ASVs were partitioned into three groups depending on their occurrence (i.e., more frequently (over-represented); less frequently (under-represented); or within the 95% confidence interval (neutrally distributed), as per neutral model predictions [42]. 

## 3. Results

### 3.1. Physico-Chemical Properties of the Coastal Sandy Sediments

**Figure 2:**
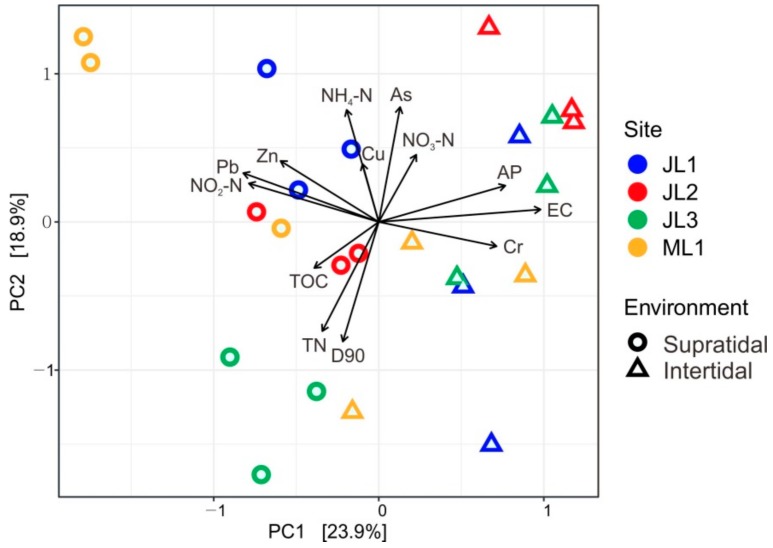
Principal component analysis (PCA) ordination biplot of the environmental factors of the intertidal and supratidal sands. Arrows in black represent environmental variables (D90: sand grain size D90 (μm); EC: electric conductivity; TOC: total organic carbon; TN: total nitrogen; NH_4_-N: ammonium-nitrogen; NO_2_-N: nitrite-nitrogen; NO_3_-N: nitrate-nitrogen; AP: available phosphorus; Cr: chromium; Cu: copper; Zn: zinc and Pb: lead). Sites JL1-JL3 located in Jinluan Bay, while site ML1 located in Maluan Bay of Dongshan Island.

In total, 13 physico-chemical parameters were determined for the coastal sandy sediments (Figure 2). The intertidal sand samples were characterized by relatively higher EC (2178.33 ± 465.60 μS/cm), higher AP content (11.86 ± 2.56 mg/kg), and higher concentration of Cr (52.08 ± 10.48 mg/kg) than those of the supratidal samples (Kruskal-Wallis test, *p* < 0.05). The supratidal sand samples had significantly higher concentrations of NO_2_-N (0.48 ± 0.20 mg/kg) and Pb (6.90 ± 1.85 mg/kg) (Kruskal-Wallis test, *p* < 0.05) compared with intertidal sand samples (Figure S1). The PCA ordination indicated that the samples from the supratidal and intertidal zones were separated from each other (Figure 2).

### 3.2. Variation in the Microbial Community Composition between the Supratidal and Intertidal Zones

**Figure 3:**
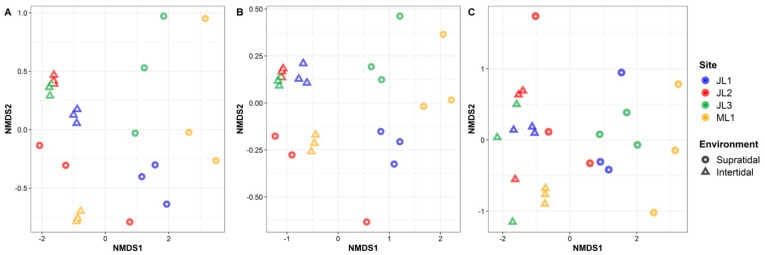
Non-metric multidimensional scaling (NMDS) ordination showing the β-diversity patterns of the (**A**) whole microbial communities, (**B**) habitats generalist, and (**C**) specialists, from coastal sandy sediments.

Overall, a total of 9860 ASVs were obtained from 24 sediment sand samples. Among them, 774 habitat generalist ASVs and 144 habitat specialist ASVs were identified that represented 15.66% and 6.34% of the total sequences, respectively. NMDS ordination analysis demonstrated that the supratidal and intertidal sand samples were clearly separated from each other, either for the whole community or for the habitat generalists-specialists, with the exception of only two of triplicate samples from supratidal zone at site JL2, that tended to cluster with the intertidal samples (Figure 3). Adonis and ANOSIM analyses further confirmed that the compositions of the whole communities and the habitat generalists-specialists were significantly different between the supratidal and intertidal samples (*p* < 0.001) (Table 1). The cluster analysis further revealed that the intertidal or supratidal sand samples from each site tended to cluster together, except for those from site JL2 (Figure S2). 

**Figure 4:**
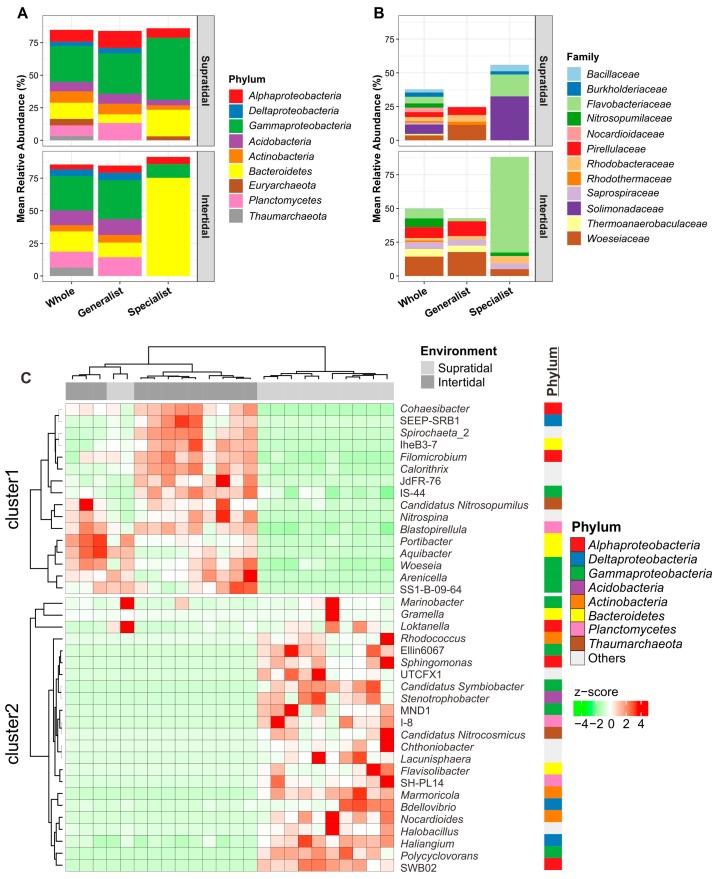
Taxonomic compositions of whole microbial communities as well as habitat generalists-specialists at the (**A**) phylum/class and (**B**) family levels. Only nine dominant phyla/classes or abundant families are shown. (**C**) Heatmap diagram showing the distribution of 39 microbial indicators (genera) in the supratidal and intertidal sands in Dongshan Island, China. Each row and column of the heat map diagram corresponds to a single indicator and sample, respectively. The row data for each indicator was z-score transformed. Dendrograms were constructed based on Spearman correlation clustering. The grey and dark grey colors in the column annotations indicate the supratidal and intertidal samples, respectively. The row annotation on the right-hand side indicates the phylum/class of each indicator.

The results of the taxonomic annotations indicated that the dominant microbial phyla/classes and families exhibited different distribution patterns between the supratidal and intertidal sands (Figure 4). For example, Gammaproteobacteria (27.51% of the whole supratidal communities), Alphaproteobacteria (9.09%), Actinobacteria (8.70%), and Euryarchaeota (4.93%) were more abundant in the supratidal than in the intertidal communities. However, an opposite pattern was observed for Bacteroidetes (15.57% of whole intertidal communities), Planctomycetes (12.14%), Acidobacteria (11.38%), Thaumarchaeota (6.47%), and Deltaproteobacteria (5.26%) (Figure 4A). Furthermore, the four most abundant families in the intertidal sands were Woeseiaceae, Pirellulaceae, Flavobacteriaceae and Nitrosopumilaceae, accounting for 14.32%, 7.98%, 7.16%, and 6.47% of all intertidal communities, respectively, but these families were less abundant in the supratidal zones (Figure 4B). Conversely, Solimonadaceae (6.90% of whole supratidal communities), Nocardioidaceae (3.39%), Rhodobacteraceae (3.07%), Burkholderiaceae (3.05%), and Bacillaceae (2.47%), had relatively higher abundances in the supratidal zone than in the intertidal zone. At the genus level, 14 of the 50 most dominant genera were more abundant in the intertidal than in the supratidal zones, while 23 genera had an opposite distribution pattern (Kruskal-Wallis test, *p* < 0.05) (Table S1). Moreover, indicator species analysis showed that 16 and 26 genera were highly associated with intertidal and supratidal communities of Dongshan Island, respectively (Figure 4C). However, it is notable that two of the triplicate samples from the supratidal zone at site JL2 clustered with the intertidal samples (Figure 4C), as described earlier by PCoA ordination (Figure 3A).

### 3.3. Relationship between Sand Microbial Communities and Environmental Factors

**Figure 5:**
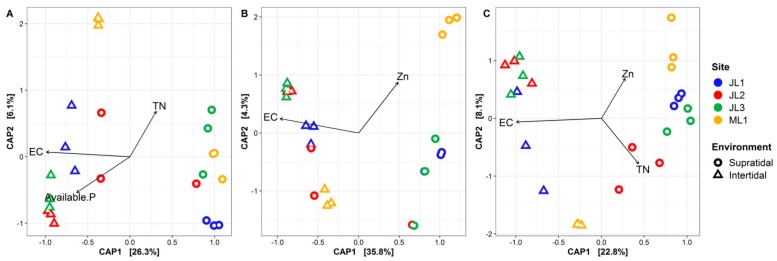
The constrained analysis of principal coordinates (CAP) based on the Bray-Curtis dissimilarity of (**A**) whole microbial communities, (**B**) habitat generalists, and (**C**) specialists from the coastal sandy sediments. Significant environmental factors were identified by using the forward selection procedure (*p* < 0.05), and then displayed in CAP ordination plots.

The CAP ordination analysis showed that EC was the most significantly contributing factor, causing the difference between the supratidal and intertidal communities (Figure 5). In addition, two other factors, AP and TN, also significantly influenced the composition of whole communities. Zn was another contributing factor in shaping the composition of habitat generalists, while Zn and TN were found to play certain roles in controlling the composition of habitat specialists. The partial db-RDA analysis indicated that the sediment physico-chemical variables alone provided more explanation for the community variation (whole communities: 10.5%; and habitat generalists: 11.8%) than spatial factors (whole communities: 6.7%; and habitat generalists: 9.7%), except for the habitat specialists (Table 2). In addition, the environmental and spatial factors jointly explained 17.3–19.1% of the variation in the microbial communities, suggesting that spatially structured environmental conditions influenced the sand microbial assemblages. Nevertheless, around 60% of the community variance could not be explained by the partial db-RDA models (Table 2).

### 3.4. The Ecological Processes Underlying the Assembly of Sand Microbial Assemblages

**Figure 6:**
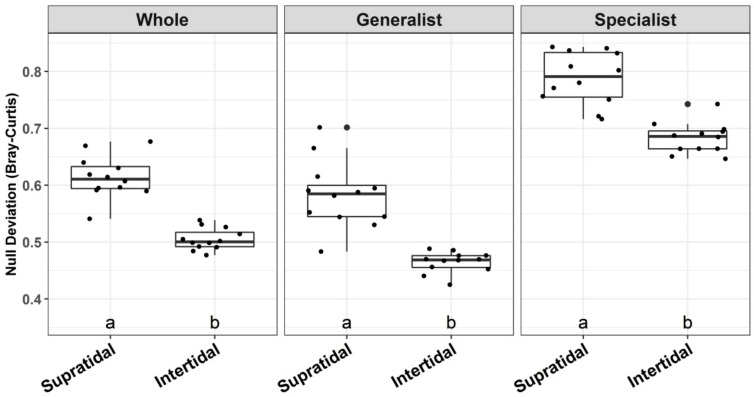
Abundance-based β-null model applied to determine the relative importance of niche and neutral processes on the assembly of whole microbial communities, as well as habitat generalists-specialists from the supratidal and intertidal sandy sediments of Dongshan Island, China.

The results of the abundance-based diversity null models showed that the β-null deviation values of the sand microbial communities tended to drift away from zero (Figure 6). The β-null deviation values were highest (supratidal: 0.79 ± 0.05; intertidal: 0.68 ± 0.03) for habitat specialist groups, and lowest for habitat generalist groups (supratidal: 0.58 ± 0.06; intertidal: 0.46 ± 0.02) in the coastal sands (Kruskal-Wallis test, *p* < 0.001). Moreover, the microbial communities from the supratidal sands harbored significantly higher β-null deviation values than their intertidal counterparts (Kruskal-Wallis, *p* < 0.001) (Figure 6). 

**Figure 7:**
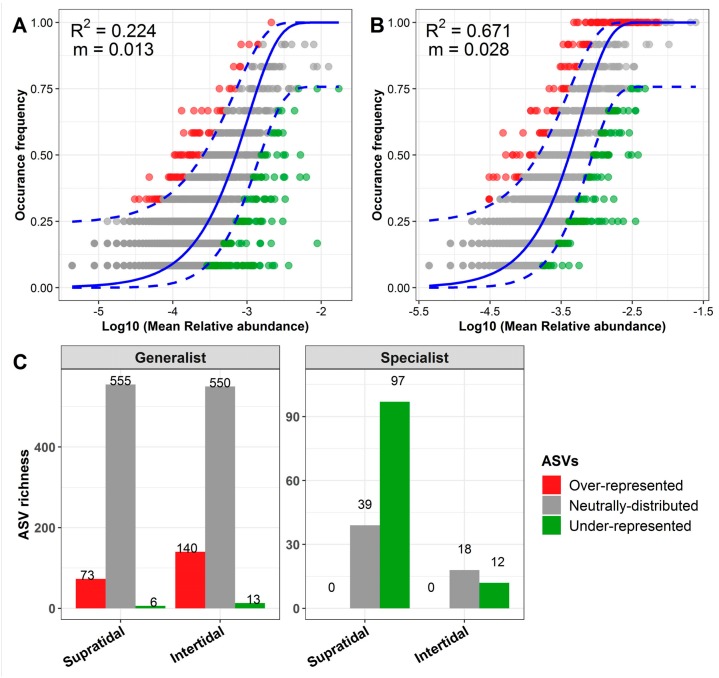
Neutral model applied to assess the effects of random dispersal and ecological drift on the assembly of (**A**) supratidal and (**B**) intertidal microbial communities. R^2^ indicates the goodness-of-fit for the neutral model. m indicates the estimated migration rate. The solid blue lines indicate the best-fit to the neutral model, and dashed blue lines represent 95% confidence intervals around the model prediction. (**C**) The bar charts depict the richness of the over-represented, neutrally distributed and under-represented ASVs in habitat generalists-specialists from the supratidal or intertidal communities.

The frequency of the occurrence of microbial ASVs in the intertidal communities (R^2^ = 0.671) showed a much better fit to the neutral model than that of the supratidal communities (R^2^ = 0.224) (Figure 7A,B). The estimated migration rates were also higher in the intertidal (m = 0.028) than in the supratidal (*m* = 0.013) communities. In addition, the ratio of the richness of the non-neutrally distributed (i.e., over- and under-represented) to neutrally distributed ASVs (i.e., the total number of over- and under-represented ASVs/the number of neutrally distributed ASVs) was higher for the habitat specialists (supratidal: 2.49; intertidal: 0.667) than for the habitat generalists (supratidal: 0.142; intertidal: 0.278), in either the intertidal or supratidal zones (Figure 7C). The ratio of the relative abundance of the non-neutrally distributed to neutrally ASVs was also higher for the habitat specialists (5.993) than for the habitat generalists (0.274) in the supratidal zone; however, in the intertidal zone, the ratio of the relative abundance of the non-neutrally distributed to neutrally ASVs was comparable for habitat generalists (0.698) and specialists (0.658) (Figure S3).

## 4. Discussion

In this study, we found that the composition of whole microbial communities was significantly different between intertidal and supratidal sands (Figure 3A). This pattern was also consistent with habitat generalists and specialists (Figure 3B,C). Such differences could be explained by the variations in the environmental conditions prevailing in intertidal and supratidal zones, especially for the EC (Figure 2 and Figure S1). The higher EC values in the intertidal zone than in the supratidal zone reflected the influence of tidal inundation of saline water. However, although interactions between site and season significantly influenced the sand microbial assemblages (Table 1), two of the triplicate samples from the supratidal zone of site JL2 tended to cluster with those from the intertidal zone (Figure S2). This is might be due to the influence of some large tidal events [7]. Nevertheless, these results are in accordance with the findings of previous studies, where salinity was described as a predominant factor in determining microbial biogeography [43,44,45,46,47]. This is possible due to the divergent adaptation of the core metabolic functions like respiration, biosynthesis of quinones and isoprenoids, glycolysis, and osmolyte transport to different salinity environments [48,49]. Thus, salinity-induced influences may inevitably lead to a niche differentiation between the intertidal and supratidal microbial taxa. As such, we observed a more distinct difference in the composition of habitat specialists between the intertidal and supratidal sands, than those of whole communities or habitat generalists (Figure 4A and B). For instance, the families *Flavobacteriaceae* (phylum *Bacteroidetes*) and *Solimonadaceae* (phylum *Gammaproteobacteria*) dominated as the habitat specialists in the intertidal and supratidal sands, respectively (Figure 4B); the former has been found as a core member in the subtidal sand grains of the North Sea [4], and is widespread in the marine environment [50]. However, species of the family *Solimonadaceae* occur mainly in soil and freshwater habitats [51]. Moreover, we found that marine thaumarchaeal *Candidatus Nitrosopumilus* [52] and soil thaumarchaeal *Candidatus Nitrocosmicus* [53] prefer to occur in the intertidal and supratidal zones (Figure 4C), respectively, providing solid evidence for the niche differentiation among the sand microbial taxa, in response to tidal seawater inundation. 

Our results indicate that, beside EC, additionally, AP, TN and Zn played a contributing role in controlling the microbial communities from the coastal sands of Dongshan Island (Figure 5). Since only AP exhibited a significant difference between the intertidal and supratidal sands (Figure S1), TN and Zn might be more likely to be responsible for the variation in the microbial communities within each environment (i.e., intertidal or supratidal zones). These findings are consistent with earlier observations, where nutrients and chemical pollutants affect the spatial distribution of sand microbial assemblages [2,6,12,54]. By using partial db-RDA analysis, we further found that a larger amount of variation in the microbial communities was explained by environmental factors (both solely and spatially structured) as compared to spatial factors (Table 2). This provides supporting evidence to the results of other microbial biogeographic studies, where the environmental filtering has been argued as a major mechanism in the assembly of sand microbial communities [7,8,12,54]. However, it is noteworthy that the spatial factors can significantly explain a proportion of the variance in sand microbial assemblages (Table 2), implying that dispersal limitations may play a certain role in shaping the sand microbiota. Although the geographic scale of our sample range was relatively small (> 6.5 km), it is still fairly possible that dispersal limitations may have occurred. Similar conclusions were made for the bacterial communities from the highly continuous environment of lentic water [55]. Moreover, given the high unexplained fraction of community variation, the effects of unmeasured abiotic/biotic factors or stochastic processes cannot be excluded [8,27,33]. For example, the contamination of polycyclic aromatic hydrocarbons could lead to changes in the composition and function of sand microbiota [12], while biotic interactions between microbial species may also have a substantial influence on sand microbial communities [27].

Numerous studies have shown that a null or neutral model-based analysis can significantly enhance our ability to explain the relative importance of different ecological processes underlying microbial community assembly [25,26,56,57,58,59]. Our results from β-diversity null models clearly support a prominent role for environmental filtering in shaping the community assembly of sand microbiota (Figure 6). More strikingly, β-diversity null model analysis revealed that the strength of environmental filtering was significantly lower for the intertidal communities than for the supratidal zone, at the whole community and population (i.e., habitat generalists-specialists) levels (Figure 6). In other words, our results suggest that the intertidal communities are more likely to be structured by neutral assembly as compared to their supratidal counterparts. These findings suggest that the neutral model was a better fit to the intertidal communities than to the supratidal microbiota, although the model fitting was not satisfactory for both the intertidal and supratidal communities (Figure 7A,B). This can be attributed to the partial stochastic community assembly of intertidal community variations through random dispersal and ecological drift [41,60]. The influence of periodic tidal inundation could enhance the possibility of microbial immigration across intertidal zones at a relatively small geographic scale [2,7,15]. A relatively higher m value was observed for the intertidal zone than the supratidal, confirming that the dispersal ability of microbial species in the intertidal zone was higher than the supratidal counterparts (Figure 7A,B). A similar finding was also reported by Yao et al. in 2019, illustrating that random dispersal played a more important role in shaping estuarine sediment microbial communities close to the sea, as compared to those far from the sea [61].

Although there is a debate regarding the relative importance of different ecological processes underlying the assembly of habitat generalists and specialists in microbial communities [17,22], our current study demonstrates that the habitat specialists of the sand microbiota were more strongly shaped by environment filtering, whereas the neutral processes affected the habitat generalist assembly to a greater extent (Figure 6). Additionally, our neutral model results revealed that a larger proportion of ASVs from habitat generalists from intertidal and supratidal sands exhibited a neutral distribution, as compared to their specialist counterparts (Figure 7C), corroborating the findings of the β-diversity null models. Since habitat generalists consist of widespread and abundant taxa in the metacommunity [17], it is highly possible that the distribution of most of the ASVs from habitat generalists was primarily driven by random dispersal [62]. Moreover, it is notable that a number of ASVs from the habitat generalists (supratidal: 73 and intertidal: 140) surpassed the specialists, hence exceeding the neutral prediction (Figure 7C). This pattern suggests that these generalists may have a competitive advantage in surviving on the dynamic coastal sands [41,42]. In contrast, a higher proportion of ASVs from habitat specialists (supratidal: 71.3% and intertidal: 40%) fell below the neutral prediction compared to their habitat generalist counterparts, highlighting the characteristic of habitat specialists that are selected for, by the specific environmental conditions of the intertidal or supratidal sands [18,22,41].

## 5. Conclusions

This study demonstrates that environmental filtering through the effects of local abiotic factors (i.e., EC and AP) play a contributing role in causing a significant variation in the composition of sand microbial communities, between the intertidal and supratidal zones. However, the relative importance of environmental filtering varied between the intertidal and supratidal microbiota, both at community and population levels. Random dispersal affected the distribution of the intertidal microbial assemblages to a greater extent, compared to their supratidal counterparts. Moreover, our third hypothesis was also confirmed: that environmental filtering dominated the assembly of habitat specialists in the coastal sand microbiota, while habitat generalists were structured by the neutral processes to some extent (e.g., random dispersal). Our study provides a novel insight into the ecological processes that govern community assembly of coastal sand microbiota at both the community and population levels. To fully understand the assembly mechanisms of the key microbial ecological groups in sandy sediments, further studies are needed at a wider spatio-temporal scale.

## Figures and Tables

**Table 1:** Significance tests of the structure of the whole microbial communities as well as habitat generalists and specialists between environments or sampling sites with Adonis and ANOSIM tests.

Group	Factor ^a^	Adonis		ANOSIM	
		R^2^	*p*	R	*p*
**Whole**	Environment	0.263	<0.001	0.669	<0.001
	Site	0.200	<0.001	0.131	0.063
	E × S	0.195	<0.001	—	—
**Habitat** **generalists**	Environment	0.337	<0.001	0.641	<0.001
	Site	0.205	<0.001	0.137	0.061
	E × S	0.194	<0.001	—	—
**Habitat** **specialists**	Environment	0.236	<0.001	0.686	<0.001
	Site	0.178	<0.001	0.062	0.178
	E × S	0.203	<0.001	—	—

^a^ Environment, supratidal and intertidal sands; Site, sampling sites; E × S, the interactive effects of environment and sampling site.

**Table 2:** Variation partitioning based on the partial db-RDA analysis using Bray-Curtis distance matrix of microbial community compositions.

	Groups	Whole	Habitat Generalists	Habitat Specialists
**Partial** **db-RDA**	Pure Env ^a^	10.5% **^b^	11.8% ***	7.4% **
	Pure Spat	6.7% **	9.7% **	8.8% **
	Shared	17.3%	19.1%	18.8%
	Total	34.5% ***	40.5% ***	35.0% ***

^a^ Pure Env, pure effect of environmental factors; Pure Spat, pure effect of geographic distance; shared, the shared effect between environmental and geographic factors. ^b^ The explained variance (adjusted R^2^) of the partial db-RDA models, reported based on a 9999 permutation test. *** *p* ≤ 0.001 and ** *p* ≤ 0.01.

## Supplementary Materials

The following are available online at https://www.mdpi.com/2076-2607/7/12/598/s1, Table S1: The relative abundance of the 50 most abundant genera in the coastal sand microbiota of Dongshan Island, Figure S1: Comparison of 13 environmental variables of the coastal sand sediments collected from Dongshan Island, China, Figure S2: Cluster analysis of the coastal sand microbiota at the whole community level, Figure S3: Bar chart depicts the relative abundance of the over-represented, neutrally distributed and under-represented ASVs in habitat generalists-specialists from the supratidal or intertidal sands.
