# Supplementary material for: Environmental Filtering Drives the Assembly of Habitat Generalists and Specialists in the Coastal Sand Microbial Communities of Southern China
Source: Microorganisms. 2019 Nov 21;7(12):598. doi: 10.3390/microorganisms7120598 (PMC6955893; doi:10.3390/microorganisms7120598)
Supplement: Supplementary file 1 [file microorganisms-07-00598-s001.pdf]

Supplementary Materials

Environmental Filtering Drives the Assembly of Habitat Generalists and Specialists in the Coastal Sand Microbial Communities of Southern China

Anyi Hu [1,*], Hongjie Wang [1,2], Meixian Cao [1,2], Azhar Rashid [1,3], Mingfeng Li [4,*] and Chang-Ping Yu [1,5]

[1] CAS Key Laboratory of Urban pollutant Conversion, Institute of Urban Environment, Chinese Academy of Sciences, Xiamen 361021, China; ayhu@iue.ac.cn (A.H.); hjwang@iue.ac.cn (H.W.); mxcao@iue.ac.cn(M.C.); azoo74@yahoo.com (A.R.); cpyu@iue.ac.cn (C.-P.Y.)

[2] University of Chinese Academy of Sciences, Beijing 100049, China

[3] Nuclear Institute for Food and Agriculture, Tarnab, Peshawar 446, Pakistan

[4] School of Biological Science and Biotechnology, Minnan Normal University, Zhangzhou 363000, China; limingfeng@mnnu.edu.cn (M.L.)

[5] Graduate Institute of Environmental Engineering, National Taiwan University, Taipei 106, Taiwan

* Correspondence: ayhu@iue.ac.cn (A.H.); Tel.: +86-592-6190582 (A.H.); limingfeng@mnnu.edu.cn (M.L.); Tel.: +86-596-2591356 (M.L.)

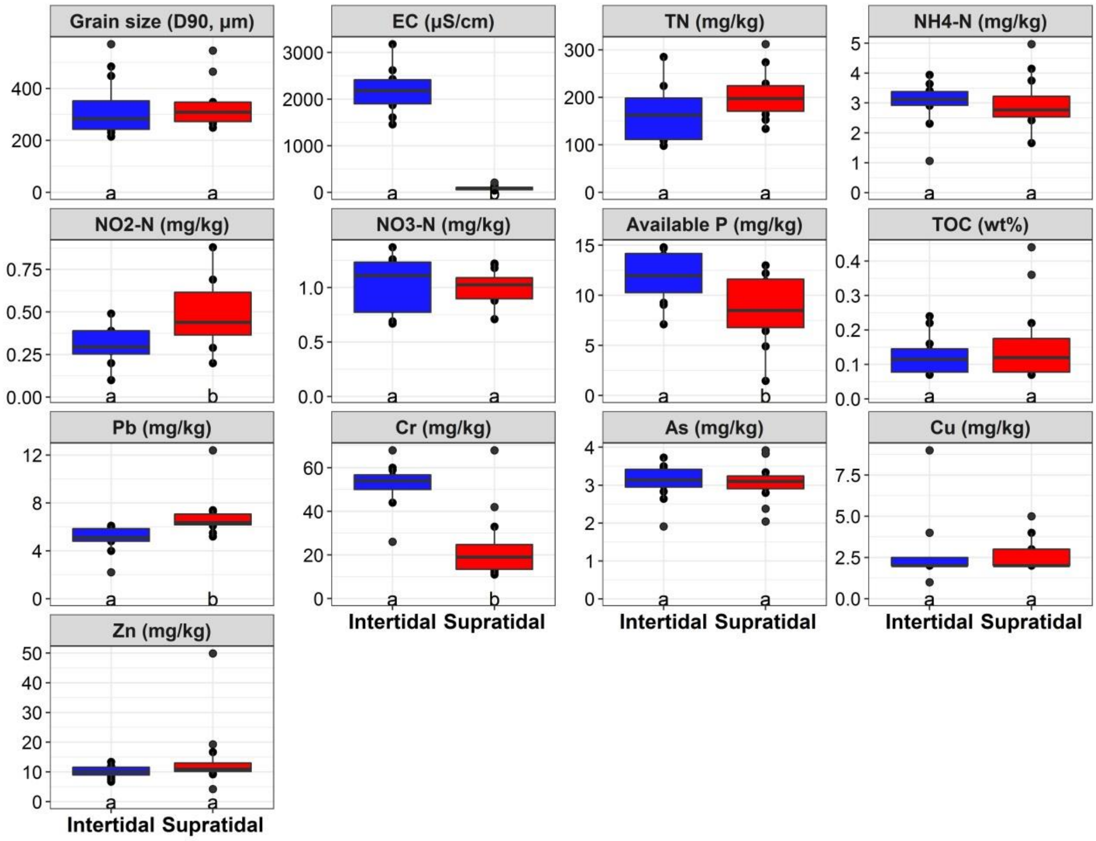

Fig. S1. Comparison of 13 environmental variables of the coastal sand sediments collected from the Dongshan Island, China. The different letters indicate significant difference between the intertidal and supratidal zones.
